# Stability analysis of reference genes for RT-qPCR assays involving compatible and incompatible *Ralstonia solanacearum*-tomato ‘Hawaii 7996’ interactions

**DOI:** 10.1038/s41598-021-97854-8

**Published:** 2021-09-21

**Authors:** Greecy M. R. Albuquerque, Fernando C. A. Fonseca, Leonardo S. Boiteux, Rafaela C. F. Borges, Robert N. G. Miller, Carlos A. Lopes, Elineide B. Souza, Maria Esther N. Fonseca

**Affiliations:** 1 Department of Agronomy, Universidade Federal Rural de Pernambuco (UFRPE), Recife, PE Brazil; 2 Departament of Academic Areas, Instituto Federal de Goiás (IFG), Águas Lindas,, GO Brazil; 3 National Center for Vegetable Crops Research, Embrapa Vegetables (CNPH), Brasília, DF Brazil; 4 Plant Pathology Department, ICB, Universidade de Brasília (UnB), Brasília, DF Brazil; 5 Department of Cell Biology, ICB, Universidade de Brasília (UnB), Brasília, DF Brazil; 6 Department of Biology, Universidade Federal Rural de Pernambuco (UFRPE), Recife, PE Brazil

**Keywords:** Plant immunity, Plant molecular biology, Biotic

## Abstract

Reverse transcription-quantitative PCR (RT-qPCR) is an analytical tool for gene expression quantification. Reference genes are not yet available for gene expression analysis during interactions of *Ralstonia solanacearum* with ‘Hawaii 7996’ (the most stable source of resistance in tomato). Here, we carried out a multi-algorithm stability analysis of eight candidate reference genes during interactions of ‘Hawaii 7996’ with one incompatible/avirulent and two compatible/virulent (= resistance-breaking) bacterial isolates. Samples were taken at 24- and 96-h post-inoculation (HPI). Analyses were performed using the ∆∆Ct method and expression stability was estimated using BestKeeper, NormFinder, and geNorm algorithms. *TIP41* and *EF1α* (with geNorm), *TIP41* and *ACT* (with NormFinder), and *UBI3* and *TIP41* (with BestKeeper), were the best combinations for mRNA normalization in incompatible interactions at 24 HPI and 96 HPI. The most stable genes in global compatible and incompatible interactions at 24 HPI and 96 HPI were *PDS* and *TIP41* (with geNorm), *TIP41* and *ACT* (with NormFinder), and *UBI3* and *PDS*/*EXP* (with BestKeeper). Global analyses on the basis of the three algorithms across 20 *R. solanacearum*-tomato experimental conditions identified *UBI3, TIP41* and *ACT* as the best choices as reference tomato genes in this important pathosystem.

## Introduction

The *Ralstonia* species complex comprises a diverse set of strains that induce bacterial wilt (BW) disease in tomato (*Solanum lycopersicum* L.) and across a large number of botanically unrelated hosts^1,2^. The *Ralstonia* complex is currently composed of the following species: *R. solanacearum* (= phylotype II from the Americas); *R. pseudosolanacearum* (= phylotypes I and III); and three subspecies of *R. syzygii* (= phylotype IV)^2–5^. The present classification is supported by both genomic and proteomic data^6,7^. Crop losses due to diseases induced by members of this complex are estimated to be around one billion dollars per year^8^.

*Ralstonia* species are not able to invade intact root surface (epidermal) tissues^9^. As a result, for these pathogens to penetrate host tissues, the presence of wounds or natural openings are required, such as partially exfoliated cells of the outer parenchyma layers at the emergence sites of secondary roots^10^. Pathogenesis of virulent *Ralstonia* strains has been described as a three-stage process^11^, including colonization of the root surface (day 1), infection of the root cortex (days 2–3), and infection of the vascular parenchyma followed by invasion of the xylem (day 3 and beyond). *Ralstonia* species synthesize virulence factors that act in facilitating infection, root xylem penetration/establishment, systemic colonization, and induction of symptoms^12^.

The inbred line *S. lycopersicum* ‘Hawaii 7996’ is considered the most stable tomato breeding source, displaying high levels of broad-spectrum resistance/tolerance to a wide array of isolates of *R. solanacearum* and *R. pseudosolanacearum*, as indicated in worldwide multi-location evaluations^13–15^. BW resistance in ‘Hawaii 7996’ is not associated with prevention of root invasion, but with the ability to restrict bacterial colonization of vascular tissues^10^. Recently, screening assays indicated that the expression of ‘Hawaii 7996’-mediated resistance/tolerance may vary according to the bacterial strain employed in trials. Challenging ‘Hawaii 7996’ against a collection of *R. solanacearum* and *R. pseudosolanacearum* isolates revealed that, in many circumstances, resistance is better classified as either phylotype-specific or strain-specific^16–18^.

In Brazil, a subgroup of *R. solanacearum* isolates was reported to cause severe breakdown of ‘Hawaii 7996’-mediated resistance^14,19^. Two of these isolates (namely ‘RS 488’ and ‘CCRMRs223’) were selected based on their ability to induce severe BW symptoms in ‘Hawaii 7996’. Conversely, a distinct *R. solanacearum* isolate (‘RS 489’) was reported as avirulent/incompatible on ‘Hawaii 7996’, but highly virulent/compatible on a set of standard susceptible cultivars^14^. From a breeding standpoint, it is of extreme interest to determine which genes are modulated (either triggered or turned off) in ‘Hawaii 7996’ by these virulent/compatible (= resistance-breaking) *R. solanacearum* isolates in comparison with the avirulent/incompatible isolates.

Reverse transcription-quantitative PCR (RT-qPCR) is one of the most reliable analytical tools to measure gene expression across a wide range of experimental conditions^20^. Robust analyses, however, require the employment of a suitable set of reference genes for transcript expression normalization^21^. In accordance with the Minimum Information for Publication of Real-Time Quantitative PCR Experiments (MIQE) guidelines^22^, normalization of expression of each target gene in relation to a reference gene is required to adjust data for variations across samples that often occur during cDNA preparation^23^.

A variety of genes have been employed as references for normalization in RT-qPCR analyses in plants^21,24^. In tomato, the major genes employed to date for expression normalization comprise beta-tubulin-4 (*TUB4*), β-6-tubulin (*TUB2*), ubiquitin (*UBQ*), glyceraldehyde-3-phosphate dehydrogenase (*GADPH*), 18S ribosomal RNA (*18S RNA*), actin (*ACT*), elongation factor 1-alpha (*EF1α*), and adenine phosphor-ribosil-transferase 1 (*APT1*)^24–32^. In addition, alternative reference genes have also been employed in RT-qPCR analyses, including the *Arabidopsis thaliana* “expressed” protein (*EXP*), TIP41-interacting protein (*TIP41*)^26^, and phytoene desaturase (*PDS*)^28^.

For the interaction *Ralstonia*-tomato, although expression stability of three genes [viz. phosphoglycerate kinase (*PGK*), alfa-tubulin (*TUB*), and *ACT*] was previously evaluated in stem tissues of inoculated plants^33^, there is so far no consistent set of reference host genes available for analysis of interactions among ‘Hawaii 7996’ and compatible and incompatible *R. solanacearum* isolates. In this study, we carried out multi-algorithm stability analysis of eight candidate reference genes for potential employment in host–pathogen interaction analysis in this important pathosystem. The precise determination of relative changes in gene expression profiles in comparative assays provides crucial and applicable information for the discovery of target genes in classical and molecular tomato breeding programs for BW resistance.

## Results

### Transcriptome RPKM values

Alleles of *ACT*, *APT*, *TUB2*, *EF1α*, *EXP*, *TIP41*, *PDS*, and *UBI3* genes in tomato were selected as candidate reference genes for RT-qPCR validation. RPKM values for these genes are summarized in Supplementary Table S1, which shows expression values with overall low standard deviations across all conditions.

### Calculation of the Cq of samples

**Figure 1:**
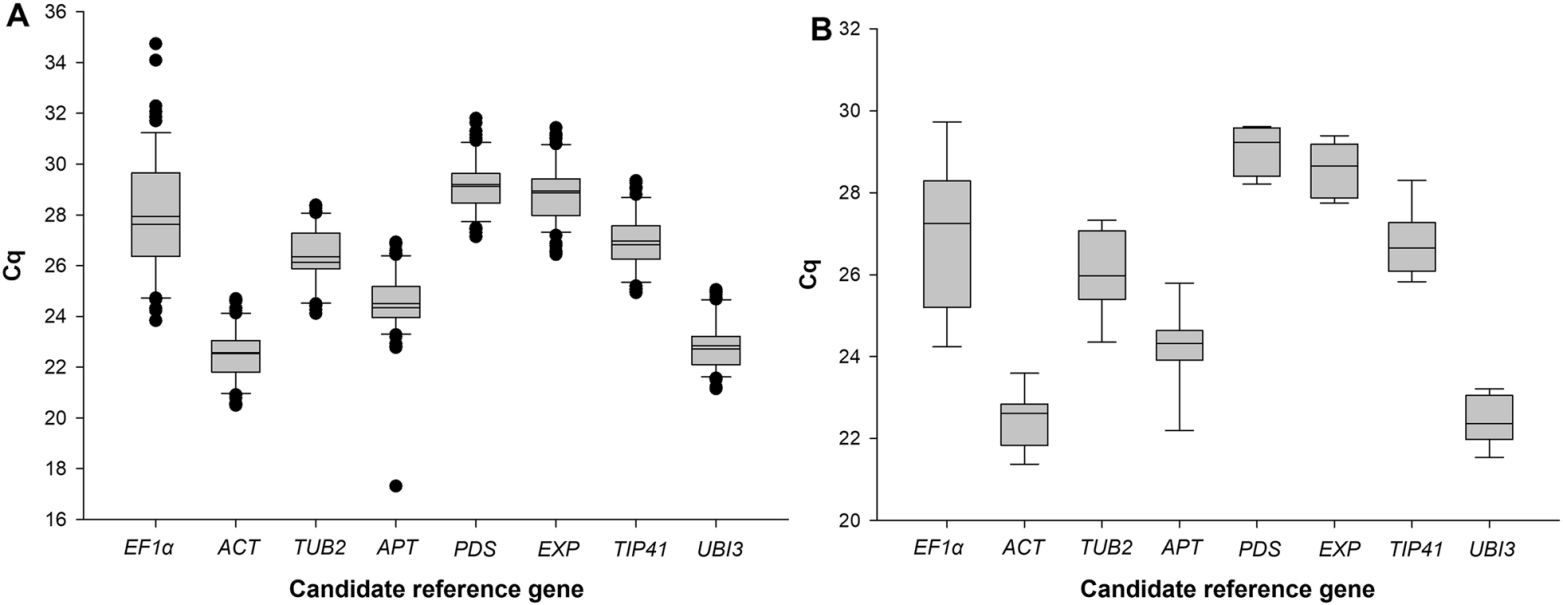
Variations in RT-qPCR Cq values of eight tomato genes/alleles [viz. actin (*ACT*), adenine-phosphoribosyl-transferase 1 (*APT*), β-2-tubulin (*TUB2*), elongation factor 1-alpha (*EF1α*), the *Arabidopsis thaliana* expressed protein (*EXP*), TIP41-interacting protein (*TIP41*), phytoene desaturase (*PDS*), and ubiquitin (*UBI3* = *UBQ*)] employed in the assays involving the pathosystem tomato ‘Hawaii 7996’ and compatible/virulent and incompatible/avirulent *Ralstonia solanacearum* isolates at different sample collection times. Box charts of Cq for each reference gene in samples of tomato ‘Hawaii 7996’ infected by *R. solanacearum* under different experimental conditions; (**Panel A**) Interactions of ‘Hawaii 7996’ with two compatible/virulent isolates compared to resistance at 24 HPI (hours post-inoculation) and 96 HPI and mock-inoculated (0 HPI). (**Panel B**) Interaction of ‘Hawaii 7996’ with an incompatible/avirulent isolate at 24 and 96 HPI compared to control mock-inoculated (0 HPI). The horizontal lines and small squares in the box plot represent median values and mean values respectively, surrounded by lower and upper boxes indicating the first and third quartile. Vertical lines indicate the value ranges.

Variation of Cq values across treatments for each gene was verified, with a Box-plot representation summarizing expression ranges and average Cq values for each gene under the two pre-defined conditions: interaction with compatible (Fig. 1A) and incompatible (Fig. 1B) *R. solanacearum* isolates. Mean Cq values for the candidate reference genes ranged between 22.50 and 29.00. Results indicated that none of the selected genes were uniformly expressed across all samples. As such, it was necessary to evaluate the candidate genes for normalization using different sets of interaction samples (analyses #01 to #20; Table 1). The set of analyses involving all contrasts among compatible and incompatible interactions (at 24 and 96 HPI) was named as the “Global Interaction”—GI (= analysis #01 in Table 1), whereas the set of analyses involving samples of the avirulent/incompatible isolate (at 24 and 96 HPI) was named as “Global Incompatible Interaction”—GII (= analysis #02 in Table 1). The results of these two analyses are summarized in Table 2. All additional specific contrasts are presented in Supplementary Table S2.

**Table 1:** Summary of analyses conducted in the development of stable sets of candidate reference genes for RT–qPCR across 20 comparisons involving interactions among the tomato inbred line ‘Hawaii 7996’, two virulent/compatible *Ralstonia solanacearum* isolates (Vir1 = RS 488 and Vir2 = CCRMRs223) and one avirulent/incompatible *R. solanacearum* isolate (Avr = RS 489).

Analyses	Contrasts	Combination of the two more stable genes**		Gene stability
		geNorm	NormFinder	BestKeeper*
#01	Vir1 × Vir2 × Avr × mock at 24 and 96 HPI	*TIP41*/*PDS*	*ACT*/*TIP41*	*UBI3*, (*PDS* = *EXP*), *ACT*, *TIP41*, *TUB2*, *APT*
#02	Avr × mock at 24 and 96 HPI	*TIP41*/*EF1α*	*EXP*/*TIP41*	*UBI3*, *TIP41*, *PDS*, *ACT*, *EXP*, *APT*, *TUB2*
#03	Vir1 × Vir2 × Avr × mock at 24 HPI	*TIP41*/*TUB2*	*APT*/*TIP41*	*APT*, *UBI3*, (*PDS* = *EXP*)
#04	Vir1 × Vir2 × Avr × mock at 96 HPI	*PDS*/*ACT*	*ACT*/*EXP*	*UBI3*, *TIP41*, *EXP*, *PDS*, (*ACT* = *APT*), *TUB2*
#05	Vir1 × Avr × mock at 24 HPI	*APT*/*ACT*	*ACT*/*TIP41*	(*ACT* = *APT*), *PDS*, (*EXP* = *UBI3*)
#06	Vir1 × Avr × mock at 96 HPI	*EXP*/*TIP41*	*ACT*/*TIP41*	*UBI3*, *TIP41*, *EXP*, *ACT*, *PDS*, *APT*, *TUB2*
#07	Vir2 × Avr × mock at 24 HPI	*TIP41/EXP*	*APT*/*TIP41*	*UBI3*, *APT*, *EXP*, (*ACT* = *TIP41*), *PDS*, *TUB2*
#08	Vir2 × Avr × mock at 96 HPI	*PDS*/*UBI3*	*ACT*/*TIP41*	*UBI3*, *PDS*, *TIP41*, *ACT*, *EXP*, *APT*, *TUB2*
#09	Vir1 × Avr × mock at 24 and 96 HPI	*UBI3/EXP*	*TIP41*/*UBI3*	*UBI3*, *EXP*, *PDS*, *ACT*, *TIP41*, *TUB2*, *APT*
#10	Vir2 × Avr × mock at 24 and 96 HPI	*UBI3*/*PDS*	*ACT*/*TIP41*	*UBI3*, *PDS*, *TIP41*, *ACT*, *EXP*, *APT*, *TUB2*
#11	Vir1 × Vir2 × mock at 24 HPI	*TIP41*/*TUB2*	*TUB2*/*APT*	….
#12	Vir1 × Vir2 × mock at 96 HPI	*TUB2/EXP*	*EXP*/*TIP41*	*UBI3*, *TIP41*, *EXP*, *ACT*, *PDS*, *TUB2*,
#13	Vir1 × Vir2 × mock at 24 and 96 HPI	*PDS*/*ACT*	*ACT*/*TUB2*	*UBI3*, *EXP*, *PDS*
#14	Vir1 × mock at 24 and 96 HPI	*APT*/*ACT*	*ACT*/*TUB2*	*UBI3*, *EXP*, *ACT*
#15	Vir2 × mock at 24 and 96 HPI	*ACT*/*PDS*	*TUB2*/*PDS*	*UBI3*, A*PT*, *PDS*, *TIP41*, *ACT*, *EXP*, *TUB2*
#16	Vir1 × Vir2 at 24 HPI	*TUB2*/*APT*	*APT*/*UBI3*	*APT*
#17	Vir1 × Vir2 at 96 HPI	*ACT*/*PDS*	*TIP41*/*UBI3*	*UBI3*, *TIP41*, *EXP*, *PDS*, *ACT*, *TUB2*, *APT*
#18	Vir1 × Vir2 × Avr at 24 and 96 HPI	*PDS*/*TIP41*	*EXP*/*TIP41*	*UBI3*, *EXP*, *PDS*, *TIP41*, *ACT*, *TUB2*
#19	Vir1 × Avr at 24 and 96 HPI	*UBI3*/*EXP*	*TIP41*/*UBI3*	(*UBI3* = *EXP*), *PDS*, (*ACT* = *TUB2*), *TIP41*
#20	Vir2 × Avr at 24 and 96 HPI	*TIP41*/*PDS*	*ACT*/*TIP41*	*PDS*, *UBI3*, *TIP41*, *EXP*, *ACT*, *TUB2*, *APT*

Biological samples were collected at 24- and 96-h post-inoculation (HPI) from entire tomato seedlings (aerial parts + root systems) inoculated with these distinct *R. solanacearum* isolates. The set of analyses involving all contrasts among compatible and incompatible interactions at 24 and 96 HPI was named as “Global Interaction”—GI (= analysis #01), whereas the set of analyses involving samples of the avirulent/incompatible isolate at 24 and 96 HPI was named as “Global Incompatible Interaction”—GII (= analysis #02).

*Gene stability ranking (higher to lower). Unlisted genes showed standard deviation values (std dev [± CP]) > 1.0.

** Code for the genes/alleles employed in the assays: actin (*ACT*), adenine–phosphoribosyl–transferase 1 (A*PT*), β–2–tubulin (*TUB2*), elongation factor 1–alpha (*EF1α*), the *Arabidopsis thaliana* expressed protein (*EXP*), TIP41–interacting protein (*TIP41*), phytoene desaturase (*PDS*), and ubiquitin (*UBI3*). = means that all genes have identical ranking position.

**Table 2:** Analyses of expression stability of eight candidate reference genes for expression normalization involving the pathosystem tomato ‘Hawaii 7996’ and virulent/compatible and avirulent/incompatible *Ralstonia solanacearum* isolates at different sampling collection times.

Gene ranking	geNorm		NormFinder		BestKeeper	
	Gene #01^a^/#02	M^b^ #01^a^/#02	Gene #01^a^/#02	Stability^c^ #01^a^/#02	Gene #01^a^/#02	Stability^d^ #01^a^/#02
1	*PDS*/*TIP*41	0.205/0.032	*TIP41*/*TIP41*	0.004/0.004	*UBI3* /*UBI3*	0.734/0.482
2	*TIP41*/*EF1α*	0.205/0.034	*EXP*/*ACT*	0.004/0.004	*PDS*/*TIP41*	0.820/0.508
3	*UBI3*/*ACT*	0.205/0.040	*ACT*/*EXP*	0.005/0.005	*EXP*/*PDS*	0.820/0.525
4	*EXP*/*PDS*	0.221/0.119	*UBI3*/*PDS*	0.008/0.009	*ACT*/*ACT*	0.853/0.531
5	*ACT*/*EXP*	0.245/0.154	*PDS*/*UBI3*	0.011/0.011	*TIP41*/*EXP*	0.862/0.584
6	*TUB2*/*UBI3*	0.291/0.176	*TUB2*/*EF1α*	0.013/0.019	*TUB2*/*APT*	0.932/0.666
7	*APT*/*TUB2*	0.392/0.292	*APT/APT*	0.016/0.021	*APT*/*TUB2*	0.942/0.807
8	*EF1α*/*APT*	0.494/0.456	*EF1α*/*TUB2*	0.022/0.021	*EF1α* /*EF1α*	1.974/1.445
BCTG	*PDS*/*TIP41* and *TIP41*/*EF1α*	…	*TIP41*/*EXP* and *TIP41*/*ACT*	…	…	…

*BCTG* best combination of two genes.

^a^Global Interaction #01 and Global Incompatible Interaction #02, as described in Table 1.

^b^M = average expression stability, calculated using the algorithm geNorm.

^c^Stability = gene expression stability calculated by intragroup and intergroup variation using the algorithm NormFinder.

^d^Stability = standard deviation (std dev [± CP]) calculated using the algorithm BestKeeper. Significant values are the ones displaying values < 1.0. All genes showed significant *p* value (= 0.001) using Pearson's correlation test.

### Analysis of gene expression stability data of the candidate reference genes

**Figure 2:**
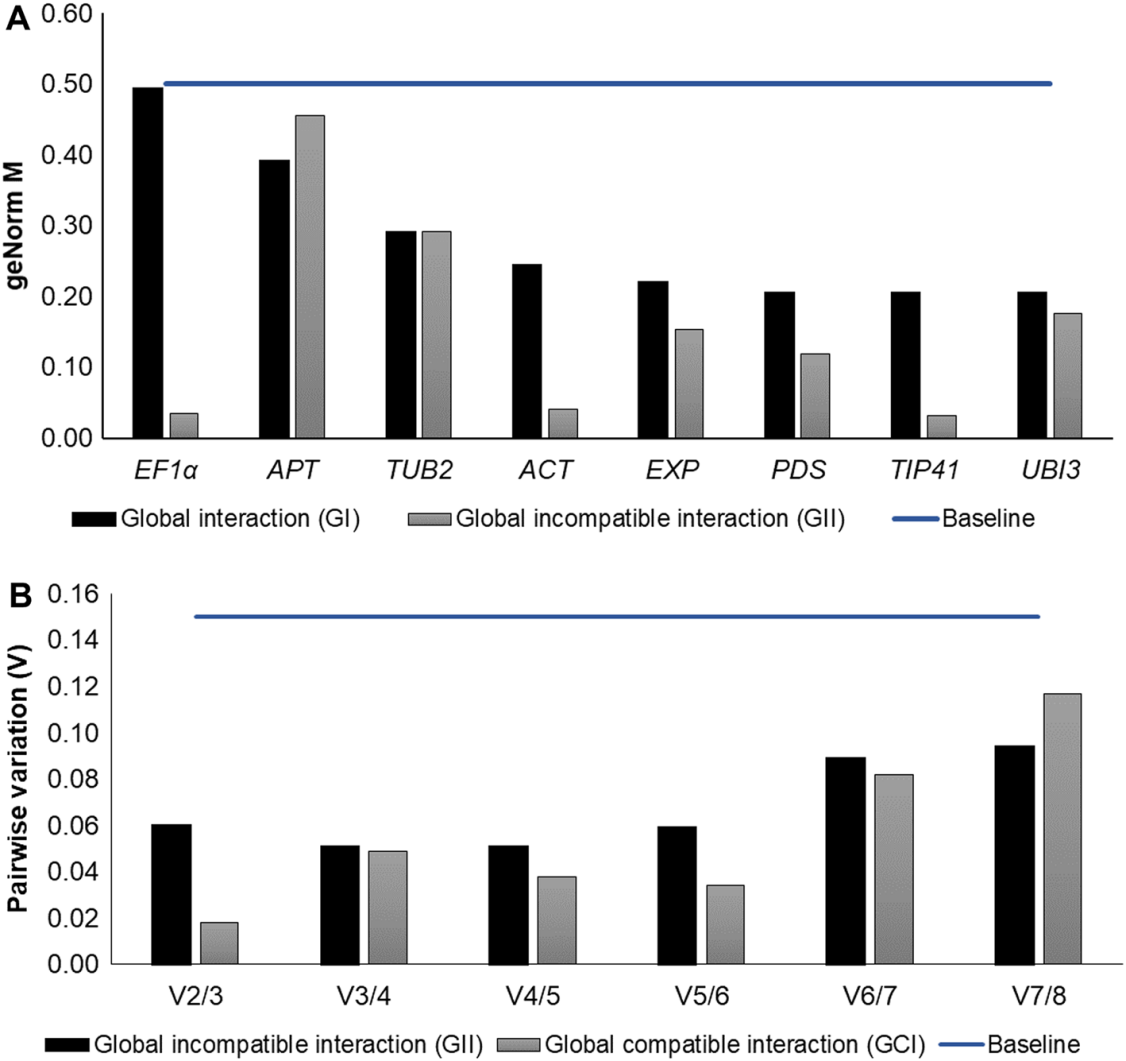
Stability analysis of eight tomato genes/alleles [viz. actin (*ACT*), adenine-phosphoribosyl-transferase 1 (*APT*), β-2-tubulin (*TUB2*), elongation factor 1-alpha (*EF1α*), the *Arabidopsis thaliana* expressed protein (*EXP*), TIP41-interacting protein (*TIP41*), phytoene desaturase (*PDS*), and ubiquitin (*UBI3* = *UBQ*)] for normalization of gene expression by RT-qPCR employed in the assays involving tomato ‘Hawaii 7996’ in interactions with incompatible/avirulent + compatible/virulent *Ralstonia solanacearum* isolates. Analyses were carried out with data from the Global Interaction #01 (Table 1) and the Global Incompatible Interaction #02 (see also Table 1) at 24- and 96-h post-inoculation (HPI) comparing to mock-inoculated control plants (0 HPI). (**Panel A**) average expression stability (M) and (**Panel B**) pairwise variation (V), calculated by geNorm algorithm in qBASE software. For data below a cutoff value of 0.15, the inclusion of additional reference genes will not contribute significantly to the normalization of gene expression data.

Expression stability and ranking of the candidate reference genes in ‘Hawaii 7996’ was determined using the algorithms geNorm, NormFinder, and BestKeeper. On the basis of geNorm analyses, all candidate reference genes showed high stability values (M < 0.5) in GI (#01) and GII (#02) (Table 2, Fig. 2). In GI, *PDS*, *TIP41*, and *UBI3* genes showed the greatest stability (M = 0.205 for all three genes), with *EF1α* the least stable of the tested genes (M = 0.49) (Table 2, Fig. 2A). In GII, *TIP41*, *EF1α*, and *ACT* genes displayed greatest stability (M = 0.032, 0.034 and 0.040, respectively), with *APT* the least stable (M = 0.456) (Table 2, Fig. 2A). *PDS* and *TIP41* were ranked as the Best Combination of Two Genes (BCTG) for GI. *EF1α* and *TIP41* were the BCTG for the GII (Table 2). In the specific interactions (analyses #03 to #20; Table 1, Supplementary Table S2) *EXP* was the most stable reference gene in analyses #07, #09, #12 and #19; *ACT* in #04, #05, #13 and #14; *PDS* in #10, #15, #17 and #20; *TUB2* in #03 and #11; *TIP41* in #06 and #18; *UBI3* in #08 and *APT* in #16.

Paired variation analysis (V) indicated that the combination of V2/3 genes met the normalization parameter requirements, with values of V < 0.15 (baseline) in GI and GII (Fig. 2), as well as in the remaining set of analyses (from #03 to #20, data not shown). V2/3 data indicated that any combination of two genes among the three most stable (identified according to their M values) was suitable for employment as reference gene pairs for expression normalization of the target genes. The inclusion of additional reference genes does not contribute significantly to the normalization of gene expression data (Fig. 2B).

**Figure 3:**
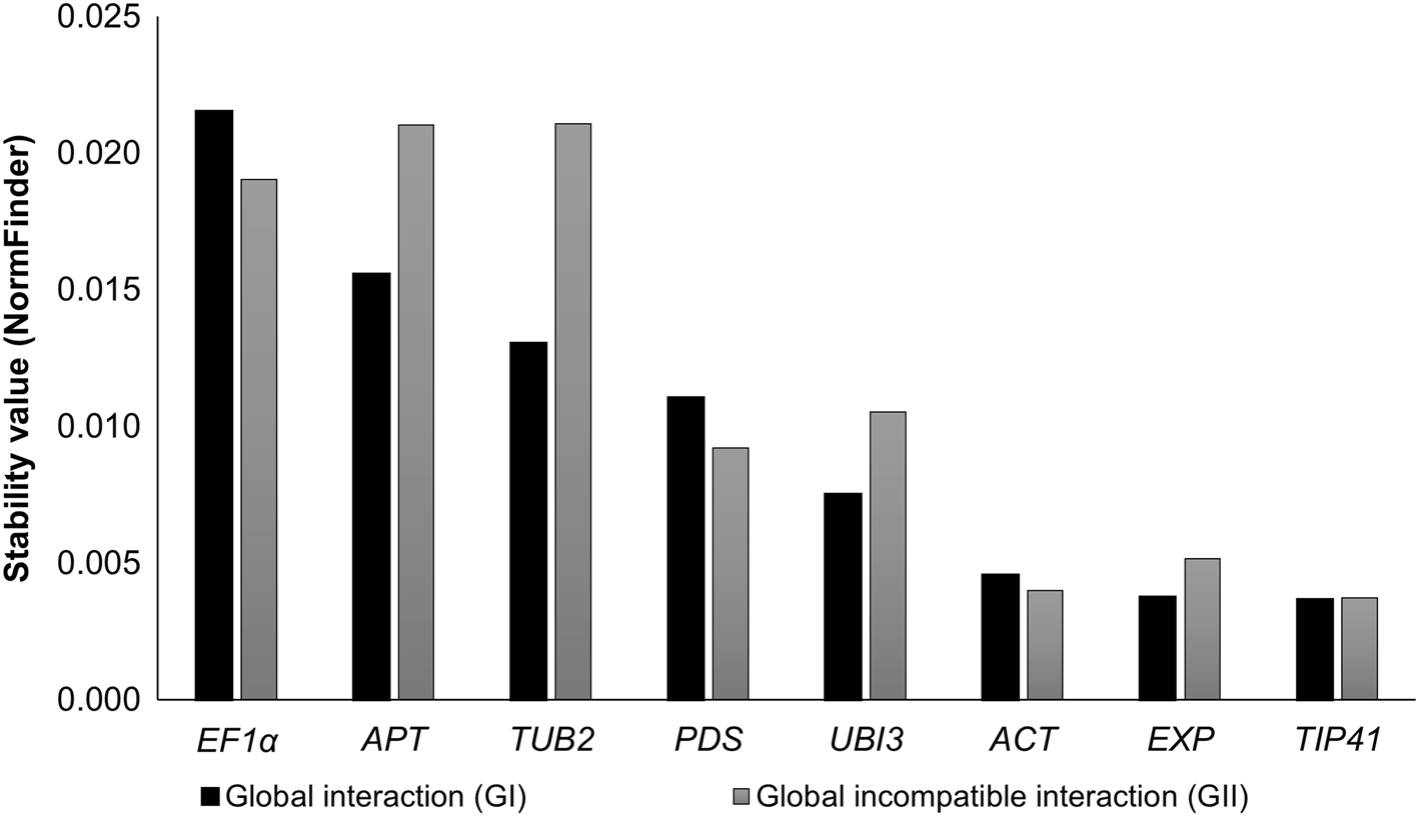
Stability values of eight tomato genes/alleles employed [viz. actin (*ACT*), adenine-phosphoribosyl-transferase 1 (*APT*), β-2-tubulin (*TUB2*), elongation factor 1-alpha (*EF1α*), the *Arabidopsis thaliana* expressed protein (*EXP*), TIP41-interacting protein (*TIP41*), phytoene desaturase (*PDS*), and ubiquitin (*UBI3* = *UBQ*)] by NormFinder algorithm for normalization of gene expression by RT-qPCR employed in the assays involving tomato ‘Hawaii 7996’ in interactions with incompatible/avirulent + compatible/virulent *Ralstonia solanacearum* isolates. Analyses were carried out with data from the Global Interaction #01 (see Table 1) and the Global Incompatible Interaction #02 (see also Table 1), at 24- and 96-h post-inoculation (HPI), and comparing to mock-inoculated control plants (0 HPI).

Although analyses with the algorithm NormFinder revealed a certain overlap with geNorm in terms of identification of the most stable genes, outputs of the algorithms varied in terms of ranking positions. For GI, *TIP41* and *EXP* (stability value = 0.004), and *ACT* (stability value = 0.005) were most highly ranked for expression stability, followed by *UBI3,* with a stability value equal to 0.008 (Table 2, Fig. 3). For GII, *TIP41* and *ACT* were the most stable genes (stability value = 0.004), followed by *EXP* and *PDS*, with stability values equal to 0.005 and 0.009, respectively (Table 2, Fig. 3). In the specific set of conditions (analyses #03 to #20; Table 1, Supplementary Table S2), *UBI3* was the most stable reference gene in analyses #12, #13, #14, #15, #17, #18 and #19; *ACT* in analysis subsets #05, #06, #08, #09 and #10; *TIP41* in subsets #03, #04, #09 and #11; *APT* in analyses #07 and #16; *EXP* in analysis #13 and *PDS* in analysis #20.

Analyses using the algorithm BestKeeper revealed all candidate genes employed in GI and GII displaying standard deviation (± CP) values lower than 1.0 (Table 2), with the exception of *EF1α*, with standard deviation (± CP) values of 1.974 and 1.445. For GI, greatest expression stability was observed with *UBI3*, *PDS*, *EXP*, and *ACT*. For GII, the most stable candidate genes were ranked as *UBI3*, followed by *TIP41*, *PDS* and *ACT* (Table 2). With this algorithm, an overlap of candidate reference genes was observed when compared to ranking positions based on geNorm and NormFinder data, especially for GI. For the specific set of analyses involving ‘Hawaii 7996’-*R. solanacearum* interactions (#03 to #20 in Table 1) *UBI3* was the gene with the lowest standard deviation [± CP] across most of the conditions (#04, #06, #07, #08, #09, #10, #11, #12, #13, #14, #15, #17, #18 and #19, Table S2). *EF1α* displayed a value higher than 1 across all the tested conditions. Interestingly, in the subset #11, none of the genes analyzed displayed adequate levels of stability according to the algorithm BestKeeper (all standard deviations > 1.0; Table S2). Subsets #13, #14 and #16 also showed numerous candidate genes with CP values above 1.0.

Overall analysis across the 20 specific conditions and on the basis of the three algorithms indicated that *UBI3, TIP41* and *ACT* can be considered as the most suitable choices as reference genes for normalization for the interaction *R. solanacearum*-tomato*.*

## Discussion

A number of previous studies have developed reference genes for normalization of RT-qPCR gene expression data in tomato, with the main genes employed to date for expression normalization comprising *TUB4*, TUB2, *UBQ*, *GADPH*, *18S RNA*, *ACT*, *EF1α*, and *APT1*. These genes were evaluated under distinct experimental conditions, which included combinations of tomato genotypes, tissue types, developmental stages, distinct abiotic stresses, as well as mycorrhizal fungus–root interactions and response to fungal, viral, and bacterial pathogens^24–31^. In addition, alternative genes have also been employed as references in RT-qPCR analyses in tomato, including *EXP*, *TIP41*^26^ and *PDS*^28^. SGN-U314153 (*CAC*) and SGN-U346908 (“*Expressed*”) genes have also been reported as an optimum combination of reference genes to normalize gene expression data during fruit development^32^, with the 2A catalytic subunit (*PP2Acs*) and SGN-U321250 (*TIP41*) the most appropriate genes for postharvest fruits subjected to electricity-induced stress^34^, and *PP2Acs* and actin (*ACT2*) identified as the most appropriate for seed tissues^35^. In a previous study of the interaction between tomato and the bacterial pathogen *Xanthomonas campestris* pv. *vesicatoria*, out of a total of 11 evaluated genes, the most suitable reference genes were identified as those coding for PHD finger family proteins and the U6 snRNA-associated protein LSm7^36^.

In this study, we evaluated eight candidate reference genes [viz. *ACT*, *APT*, *TUB2*, *EF1α*, *EXP*, *PDS*, *TIP41*, and *UBI3*] for potential RT-qPCR analysis of gene expression during interactions among *R. solanacearum* isolates and *S. lycopersicum* ‘Hawaii 7996’, a genotype considered thus far the best breeding source of resistance to this group of pathogens. In addition, we were able to dissect interaction expression profiles that represented sampling of the whole plant, i.e., a bulk of leaf, root, and stem tissues, at 24 HPI and 96 HPI. Moreover, the reference genes described here were found to be appropriate for investigation of host gene expression during interaction with *R. solanacearum* isolates with contrasting virulence profiles (compatible versus incompatible against this resistant tomato line). Hence, our experimental conditions were distinct and more comprehensive than the single previous study dealing with the tomato-*R. solanacearum* pathosystem^33^. In this former study, RNA expression stability of the housekeeping genes *PGK*, *TUB*, and *ACT* in stem tissues in the tomato cultivar ‘King Kong 2’ (which displays only moderate levels of resistance) was evaluated in conjunction with a silicon priming treatment conducted prior to infection with a highly virulent *R. solanacearum* strain (race 1, biovar 3).

Our results were consistent, and at least one pair of selected reference genes were appropriate for gene expression analysis in each of the 20 possible sample combinations. Sampling time, as well as pathogen isolate, were considered in reference gene development, with ranking of stably expressed reference genes consistently varying across the treatments. These observations reinforce the importance of validating reference genes for specific sets of experimental conditions, especially in the case of the tomato-*R. solanacearum* pathosystem. Primer sets developed here were found to be appropriate for investigation of host gene expression at 24 and 96 HPI, which are crucial time points in terms of *R. solanacearum* infection and pathogenesis. Previous histological investigation of tomato infection with compatible *Ralstonia* strains reported initial colonization of root surfaces and intercellular cortical spaces at 24 HPI, followed by cortical infection of vascular parenchyma and xylem from 72 HPI onwards^11^.

Normalization of RT-qPCR experiments ensures that results are both statistically significant and biologically meaningful^32^, with inappropriate reference genes potentially resulting in misinterpretation of gene expression data and erroneous conclusions^37^. Furthermore, in specific cases for bacterial pathogens, the most suitable reference genes have been shown to be dependent upon the experimental conditions employed for calibrating RT-qPCR analyses. For this reason, reference genes were validated for specific virulent and avirulent ‘Hawaii 7996’-*R. solanacearum* interactions at different times after inoculation. Results revealed that no single algorithm was appropriate to exclusively determine candidate reference gene expression stability in ‘Hawaii 7996’ in these interactions. Additionally, as each algorithm for analysis of expression stability is based on a different statistical method, differences in gene stability ranking often occur when analyzing datasets. As such, a standard procedure is to consider methods as complementary, with outcomes equally weighted and combined into a ‘consensus’ ranking. Here, the most stable genes varied according to the software employed, with the exception of the *TIP41* gene. Overall, the housekeeping and ‘classical’ genes performed as well as the ‘nonclassical’ genes. A certain degree of overlap in the selection of the two most stable reference genes was observed across the algorithms.

Although expression stability analyses in the same experimental treatment revealed differences when employing algorithms geNorm and NormFinder, the most highly stable pairs of genes, as well as the same single gene, were identified by both algorithms in 80% of the analyses. For example, when considering GI and GII, *TIP41* was consistently identified as the most stable gene across both experiments with both algorithms, although the second gene was quite divergent. Identical stability ranking was similarly reported^28^, for two out of ten reference genes examined for normalization of gene expression by RT-qPCR in the tomato-begomovirus interaction using geNorm, NormFinder, and BestKeeper algorithms. In this scenario, the employment of two or more algorithms should be endorsed as a standard methodological procedure in order to obtain more precise assessments of reference gene stability^26,38^. BestKeeper is an algorithm that identifies the most stable reference gene based on pairwise correlation analysis of candidate genes, calculating the geometric mean of the most stable. As a default, reference genes displaying a variation in amount of starting material by a factor of two or more are considered unstable^39^. This equates, in a PCR reaction with an amplification rate of two (100% reaction efficiency), to genes whose Cq values display standard deviation (± CP) values > 1. Given that in biological samples the reaction efficiency is rarely 100%^40^, the default setting of BestKeeper might be inappropriate for in vivo samples, limiting applicability to restricted experimental conditions^41^. Examples of discrepancies in rankings of gene stability data with BestKeeper in relation to the algorithms GeNorm and NormFinder has been documented in recent analyses of plant gene expression^42^. In our analysis, BestKeeper determined CP < 1 values for most genes, which were ranked according to their stability. However, samples extracted at 24 HPI appear to show higher CP values, as observed in groups #3, #5, #7, #11 and #16, with no stable gene determined for group #11 (Table S2). The groups where samples extracted at 24 and 96 HPI were analyzed together also showed a tendency for higher CP values, as observed in groups #13 and #14. This may be related to the initial level of gene expression in the plant biological response, with RPKM values from in silico data showing similar expression levels in these genes to the mock 0 HPI sample, decreasing at 96 HPI (Table S1).

Across all specific sets of *R. solanacearum*-tomato ‘Hawaii 7996’ interactions, *TIP41, EXP, UBI3* and *ACT* outperformed all other genes in terms of expression stability. Interestingly, in contrast to our study, the *ACT* gene (actin 4; Solyc04g011500) was previously reported as displaying unstable expression^33^, in particular during the early infection phase. Here, we employed a different *ACT* gene (= Actin 51; SolycSolyc11g005330.2.1), using a distinct primer pair, and sampling both aerial and root tissues. This *ACT* allele displayed a stable level of mRNA expression across all specific sets of ‘Hawaii 7996’-*R. solanacearum* interactions, highlighting the importance of developing reference genes for normalization with specific alleles that are suitable for both plant genotype and imposed experimental conditions. In addition, it is recognized that the information from paired gene variation is required to perform accurate normalization assays^28^. Hence, according to our results, any of the combinations of pairs of genes *PDS*/*TIP41*, *PDS*/*UBI3*, or *TIP41*/*UBI3* may be employed as reference genes for GI. Similarly, *TIP41*/*EF1α*, *TIP41*/*ACT*, or *EF1α* /*ACT* were found to be the most appropriate combinations of reference genes for GII.

We employed RNAseq transcriptome data to double-check the stability of the candidate reference genes for the *R. solanacearum*-tomato ‘Hawaii 7996’ pathosystem based on RPKM analysis. We found that the transcriptome per se functioned as a reliable predictor of reference gene stability, with all the reference genes tested via RT-qPCR, including the *PDS* gene (which showed low RPKM values), showing stable expression across the treatments.

In summary, this study is the first comprehensive investigation into validation of appropriate reference genes for one of the most economically and biologically relevant tomato pathosystems. Stable gene combinations were identified which are appropriate for accurate examination of target gene expression via RT-qPCR. The emergence of novel *R. solanacearum* isolates that are able to break down ‘Hawaii 7996’-derived resistance (which is one of the few effective sources of resistance in tomato breeding programs) is a major threat that will require advanced biotechnological approaches to develop genetic solutions for control of these bacterial variants. In this scenario, well-designed transcriptomic and RT-qPCR assays will play a major role in molecular breeding programs. The employment of these sets of genes with stable expression will have crucial methodological relevance for the identification of key genes of interest for development of tomato cultivars with stable and durable BW resistance.

## Methods

### Plant material and inoculation assays with contrasting *R. solanacearum* isolates

Whole seedlings (all aerial organs plus root system) of the tomato inbred line ‘Hawaii 7996’ were employed in the bioassays. The use of plants in the present study complies with international, national and/or institutional guidelines. All plant material was provided by Embrapa. Three *R. solanacearum* isolates with contrasting virulence/compatibility patterns in relation to ‘Hawaii 7996’ were employed in the study. The isolates CCRMRs223 (from tomato, Brazil, sequevar IIA-63)^19^ and ‘RS 488’ (from tomato, Brazil, sequevar IIB-1) are able to induce severe wilt symptoms in ‘Hawaii 7996’^14^. Contrasting results were obtained with the isolate ‘RS 489’ (from tomato, Brazil, sequevar IIA-50). ‘RS 489’ is known to be avirulent/incompatible on ‘Hawaii 7996’^14^, but is highly virulent/compatible against a set of susceptible tomato cultivars/lines (viz. ‘Caline IPA-6’, ‘LS-391’, and ‘hybrid TY-2006’)^14,18,19^. Seedlings (30 days after sowing, displaying four fully-expanded leaves) were separately inoculated with the *R. solanacearum* isolates via root spraying, with bacterial suspensions adjusted to 1 × 10^8^ CFU mL^−1^. Subsequently, inoculated plants were transplanted to 500 mL pots filled with a sterile substrate mixture. Symptoms were observed in susceptible cultivars 5–7 days after inoculation, demonstrating the adequacy of the inoculation procedure.

### Experimental design and biological samples

Experiments were carried out under greenhouse conditions at CNPH (Brasília, DF, Brazil) in a completely randomized design, with three biological replicates of ten plants each. A mix of root and entire aerial plant parts were collected from ten tomato seedlings for each treatment. Plant material was flash frozen in liquid nitrogen and stored at − 80 °C until RNA extraction. The biological treatments consisted of one tomato line/three bacterial isolates and sampling at 24 and 96 HPI. Sterile water-treated plants were collected at 0 HPI, representing the mock-inoculated control treatment (Table 1).

### RNA extraction and cDNA synthesis

Total RNA was extracted from a liquid nitrogen-derived macerate of 1 g of tissue mix (root and stems) of the ten tomato seedlings of each replicate using the TRI Reagent protocol (Sigma Aldrich, St. Louis, MO, USA), with final resuspension in nuclease-free water. Extractions were performed in triplicate for each biological treatment. After extraction, RNA was treated with DNase1 and the RNeasy MinElute Cleanup Kit (Qiagen, Hilden, Germany), to remove DNA and PCR inhibitors. Quantification and quality analysis of RNA samples was conducted on an Agilent 2100 Bioanalyzer (Agilent Technologies, Santa Clara, CA, USA) using an RNA 6000 Nano LabChip kit (Agilent, Germany). A sample of each repetition/treatment with high quality extracted RNA [260/280 ratio > 1.98 and RNA integrity number (RIN) ≥ 8.0] was then employed for cDNA synthesis. A total of 1 µg of each RNA sample was employed in the synthesis of first strand cDNA using the SuperScript IV VILO Kit cDNA Synthesis kit (Thermo Fisher Scientific, San Jose, CA, USA). For each of the seven conditions (one tomato line/three bacterial isolates/collection times of 24 HPI, 96 HPI and mock at 0 HPI), cDNAs of the three biological replicates were synthesized, comprising a total of 21 samples. Each cDNA was synthesized under the RT conditions (= positive reverse transcription) and NRT (= negative reverse-transcription), in order to rule out contamination of RNA with genomic DNA. Efficiency of cDNA synthesis was determined via agarose gel electrophoresis (1%). Individual samples were employed in both transcriptomic library synthesis and RT-qPCR validation.

### Next-generation RNA sequencing (RNA-Seq)

Next-generation RNA-Seq was performed to investigate the transcriptional panorama of the multiple interactions of ‘Hawaii 7996’ and *R*. *solanacearum* isolates, using the same samples employed in RT-qPCR analyses. In these assays, RNA-Seq libraries were prepared using a pool of three replicates of each of the seven treatments in an equimolar ratio, with a final size selection of 400–500 bp. After quality control confirmation, seven RNA-Seq barcoded and pooled libraries were paired-end-sequenced (2 × 100 bp) in a single lane on a HiSeq 2500 Genome Analyzer (in rapid run mode) (Illumina Inc, San Diego, CA, USA). Base calls were assigned using Illumina Real-Time Analysis software (Ver. 1.17.20) and binary base call (BCL) files were converted to a flat-file format (qseq.txt) using Illumina BCL Converter software (Version 1.9.4). Qseq.txt files were de-multiplexed to single sample FASTQ files using the de-multiplexer software of the Centro de Genômica Funcional from ESALQ-USP, Piracicaba, SP, Brazil. Raw RNA-Seq reads (FASTQ) were processed and analyzed using the Lasergene Genomics Suite (DNAStar, Madison, WI, USA). The paired-end reads were assembled with SeqMan NGen (Ver. 15), using default parameters and aligned to the reference tomato genome (version SL.2.50) and ITAG2.4 with publicly available annotations (https://solgenomics.net/jbrowse_solgenomics/). ArrayStar (Version 15; DNAStar, Madison, WI, USA) was employed for normalization and statistical analysis of differential gene expression of mapped paired-end reads, using the default parameters. Expression data quantification and normalization were calculated using the RPKM (reads per Kb per million) value for each gene^43^.

### Selection of candidate reference genes and design of allele-specific primers

A total of eight tomato genes/alleles were selected as potential reference genes [viz. actin (*ACT* = Solyc11g005330.2); adenine-phosphoribosyl-transferase 1 (*APT* = Solyc04g077970.4); β-2-tubulin (*TUB2* = Solyc10g086760.2); elongation factor 1-alpha (*EF1α* = Solyc06g009970.3); the *A. thaliana* expressed protein (*EXP* = Solyc07g025390.4); TIP41-interacting protein (*TIP41* = Solyc10g049850.3); phytoene desaturase (*PDS* = Solyc11g007370.3), and ubiquitin (*UBI3* = UBQ = Solyc12g098940.2)]. Corresponding gene-specific primer sequences are listed in Table 3. For a subgroup of these selected genes, primers were previously validated in distinct pathosystems involving tomato, as described in the literature (Table 3). Additional in silico amplification assays using these primer pairs were performed using sequence information derived from the transcriptome data in the present study.

**Table 3:** Supporting information on the selected primer pairs and amplification profiles of the candidate reference genes for reverse transcription-quantitative PCR (RT-qPCR) normalization involving the pathosystem tomato ‘Hawaii 7996’ and compatible/virulent and incompatible/avirulent *Ralstonia solanacearum* isolates.

Gene symbol	Gene name	Sequence 5′–3′ (F and R)	PCR amplicon length (bp)	Amplification efficiency (%) ± SD	References
*ACT*	Actin	CGGTGACCACTTTCCGATCT	62	103.0 ± 0.0196	^25^
		TCCTCACCGTCAGCCATTTT			
*APT*	Adenine-phosphoribosil-transferase1	GAACAGACAAGATTGAGATGCATGTA	60	94.3 ± 0.0222	^25^
		CCACGAGGGCACGTTCA			
*TUB2*	β-2-tubulin	TTGGTTTTGCACCACTGACTTC	84	95.5 ± 0.0232	^25^
		AAGCTCTGGCACTGTCAAAGC			
*EF1α*	Elongation factor 1-α	GATTGACAGACGTTCTGGTAAGGA	67	86.0 ± 0.0204	^25^
		ACCGGCATCACCATTCTTCA			
*EXP*	Expressed sequence	GCTAAGAACGCTGGACCTAATG	183	94.4 ± 0.0250	^26^
		TGGGTGTGCCTTTCTGAATG			
*PDS*	Phytoene Desaturase	GCCGATTGTGGAACATATTGAGTC	91	91.4 ± 0.0193	^28^
		GACACTTCCATCCTCATTCAGCTC			
*TIP41*	TIP41-interacting protein	ATGGAGTTTTTGAGTCTTCTGC	235	92.3 ± 0.0162	^26^
		GCTGCGTTTCTGGCTTAGG			
*UBI3*	Ubiquitin 3	AGAAGAAGACCTACACCAAGCC	119	95.1 ± 0.0280	^28^
		TCCCAAGGGTTGTCACATACATC			

### Evaluation of amplification efficiency

RT-qPCR reactions were performed using the Universal SYBR Green detection system (Invitrogen, Carlsbad, CA, USA). Amplifications were conducted using 0.1 mL MicroAmp Fast Optical 96-well reaction plates with barcodes (Applied Biosystems, Foster City, CA, USA), sealed with MicroAmp™ Optical Adhesive Film (Applied Biosystems). Gene expression was quantified by the ∆∆Ct method^44^. Reactions were performed on a StepOne™ 7500 fast Real-Time PCR System (Applied Biosystems) at the Plant-Pest Interaction Laboratory at the Universidade de Brasília (UnB), Brasília-DF, Brazil. The reaction mix consisted of iTaq Universal SYBR Green Supermix (Bio-Rad, Hercules, CA, USA) (1X), forward and reverse primers (0.2 µM each), 1:20 cDNA (2.0 µL) and adjustment with nuclease-free water to a final volume of 10 µL. Amplification conditions were as follows: initial hold at 52 °C for 2 min, denaturation at 95 °C for 10 min, followed by 40 cycles of 95 °C for 15 s and 60 °C for 1 min. Each gene was analyzed in three biological replicates, with technical triplicates included per repetition. Primer specificity was also double-checked by confirming the presence of a single peak on melting curves in RT-qPCR analysis (Supplementary Figure S1), together with agarose gel analysis of all amplicons obtained via RT-PCR. Supplementary Figure S2 shows an example of specificity for the tested gene *UBI3*.

### Stability analysis and validation of candidate reference genes

Twenty comparisons were analyzed amongst the seven biological samples from the ‘Hawaii 7996’-*R. solanacearum* pathosystem (Table 1). These sets comprised one incompatible (one avirulent *R. solanacearum* isolate and ‘Hawaii 7996’) and two compatible (viz. two virulent*/*compatible *R. solanacearum* isolates and ‘Hawaii 7996’) interactions, as well as the mock-inoculated samples at 24 and 96 HPI, with three replicates (Table 1). Analyses were based on the values ​​of the quantification cycle (Cq), calculated according to the efficiency of the reaction for each primer, estimated using LinReg software^45^. The Cq value represents the number of amplification cycles during the exponential PCR amplification phase that are required to reach a default threshold value for detection. The Cq average and variation for each gene were analyzed with Box-plot graph in the program SigmaPlot v. 12.5 (Systat Software, San Jose, CA, USA). Validation of the candidate reference genes was performed using three different analytical algorithms: BestKeeper^46^ and NormFinder^47^ in Excel, and geNorm using qBASE software, version 3.0 (Biogazelle, Zwijnaarde, Belgium), according to the default parameters of the software and as previously described^48^.

### Performance of the primers for each candidate reference gene

Expression stability was examined across ‘Hawaii 7996’ samples, at different times after inoculation and with compatible and incompatible *R. solanacearum* isolates. Melting curve analysis was performed by RT-qPCR after 40 cycles of amplification. The specificity of the primer pairs for each candidate gene was confirmed through both visualization of single amplicons after standard PCR assays, together with RT-qPCR dissociation curve analysis and observation of single peaks, following calculations with LinReg PCR (Supplementary Figure S1). RT-qPCR amplification efficiencies from the standard curves ranged from 86 to 101% (Table 3).

## Supplementary Information

Supplementary Table S1.

Supplementary Table S2.

Supplementary Figure S1.

Supplementary Figure S2.

## Data Availability

All data generated and analyzed during the study is included in the published article and Supplementary Information files.
