# Supplementary material for: Stability analysis of reference genes for RT-qPCR assays involving compatible and incompatible Ralstonia solanacearum-tomato ‘Hawaii 7996’ interactions
Source: Sci Rep. 2021 Sep 21;11:18719. doi: 10.1038/s41598-021-97854-8 (PMC8455670; doi:10.1038/s41598-021-97854-8)
Supplement: Supplementary file 1 — Supplementary Table S1. [file 41598_2021_97854_MOESM1_ESM.docx]

**Supplementary Table S1.** Relative expression levels of eight tomato genes/alleles: [actin (A*CT*), adenine–phosphoribosyl–transferase 1 (*APT*), β–2–tubulin (*TUB2*), elongation factor 1–alpha (*EF1α*), the *Arabidopsis thaliana* expressed protein (*EXP*), TIP41–interacting protein (*TIP41*), phytoene desaturase (*PDS*), and ubiquitin (*UBI3* = *UBQ*)] from whole plant tissues analyzed via RNAseq in assays involving the pathosystem tomato ‘Hawaii 7996’ and compatible/virulent and incompatible/avirulent *Ralstonia solanacearum* isolates.  The expression level of each gene was calculated using the RPKM (reads per Kb per million) value (Mortazavi et al., 2008). The signal values for each experiment were divided by the total bases of target sequence divided by one thousand; the resulting number was then divided by the total number of mapped reads divided by one million (Lasergene Ngene, DNAStar, Madison, WI, USA).

|  |  | | **RPKM*** | | | | | | | |
| --- | --- | --- | --- | --- | --- | --- | --- | --- | --- | --- |
| **Gene code** | **Gene/allele code** | **Sol Genomics gene/allele code** | **Mock**  **0 HPI** | **CCRM**  **Rs223**  **24 HPI** | **RS 488**  **24 HPI** | **RS 489**  **24 HPI** | **CCRM**  **Rs223**  **96 HPI** | **RS 488**  **96 HPI** | **RS 489**  **96 HPI** | **Std.**  **dev.** |
| *ACT* | LOC101262163 | Solyc11g005330.2 | 11335 | 10912 | 10791 | 9355 | 8502 | 9638 | 7737 | 1337 |
| *EF1α* | LOC101244084 | Solyc06g009970.3 | 10801 | 10591 | 11115 | 9122 | 7109 | 7430 | 6958 | 1847 |
| *APT* | LOC101260722 | Solyc04g077970.4 | 4077 | 4240 | 4062 | 3189 | 4148 | 3904 | 3975 | 350 |
| *UBI3* | LOC101248559 | Solyc12g098940.2 | 1950 | 2685 | 2387 | 2267 | 1943 | 2048 | 2141 | 268 |
| *TUB2* | LOC101252240 | Solyc10g086760.2 | 1177 | 877 | 946 | 695 | 550 | 580 | 412 | 266 |
| *TIP41* | LOC101255442 | Solyc10g049850.3 | 727 | 835 | 678 | 630 | 493 | 515 | 425 | 145 |
| *EXP* | LOC101263039 | Solyc07g025390.4 | 147 | 134 | 144 | 110 | 95 | 91 | 82 | 27 |
| *PDS* | LOC101250467 | Solyc11g007370.3 | 31 | 20 | 13 | 14 | 8 | 11 | 0 | 10 |

*Gene expression values of the mock–inoculated control (= mock) at 0 hours post-inoculation (hpi), incompatible treatments of ‘Hawaii 7996’ inoculated with the incompatible/avirulent RS 489 strain at 24 hpi and 96 hpi as well as ‘Hawaii 7996’ inoculated with two compatible/virulent strains (RS 488 and CCRMRs223) at 24 hpi and 96 hpI.
