# Supplementary material for: Stability analysis of reference genes for RT-qPCR assays involving compatible and incompatible Ralstonia solanacearum-tomato ‘Hawaii 7996’ interactions
Source: Sci Rep. 2021 Sep 21;11:18719. doi: 10.1038/s41598-021-97854-8 (PMC8455670; doi:10.1038/s41598-021-97854-8)
Supplement: Supplementary file 2 — Supplementary Table S2. [file 41598_2021_97854_MOESM2_ESM.docx]

**Supplementary Table S2.** Stability expression analysis for candidate reference genes in tomato line ‘Hawaii 7996’. Experimental treatments contrasted in terms of different specific sets of two compatible/virulent (Vir) isolates and one incompatible/avirulent (Avr) *Ralstonia solanacearum* isolate, with samples collected at two distinct time points (24- and 96-hours post-inoculation [hpi]), and comparisons made to mock-inoculated (0 HPI) controls (for details see Experiments #03 to #20, Table 1).

|  | **geNorm** | | **NormFinder** | | **BestKeeper** | |
| --- | --- | --- | --- | --- | --- | --- |
| **Gene Ranking** | **Gene** | **M^1^** | **Gene** | **Stability**  **Value^2^** | **Gene** | **Std dev [±CP]^3^** |
| **Analysis #03:** Vir1 *x* Vir2 *x* Avr *x* mock at 24 HPI | | | | | | |
| 1 | *TUB2* | 0.195 | *TIP41* | 0.004 | *APT* | 0.927 |
| 2 | *TIP41* | 0.212 | *APT* | 0.004 | *UBI3* | 0.974 |
| 3 | *APT* | 0.247 | *ACT* | 0.006 | *PDS* | 0.990 |
| 4 | *ACT* | 0.274 | *EXP* | 0.008 | *EXP* | 0.992 |
| 5 | *UBI3* | 0.360 | *UBI3* | 0.010 | *ACT* | 1.010 |
| 6 | *EXP* | 0.562 | *TUB2* | 0.011 | *TUB2* | 1.051 |
| 7 | *EF1α* | 0.706 | *PDS* | 0.013 | *TIP41* | 1.101 |
| 8 | *PDS* | 0.842 | *EF1α* | 0.023 | *EF1α* | 2.278 |
| **Analysis #04:** Vir1 *x* Vir2 *x* Avr *x* mock at 96 HPI | | | | | | |
| 1 | *ACT* | 0.179 | *TIP41* | 0.004 | *UBI3* | 0.408 |
| 2 | *PDS* | 0.186 | *ACT* | 0.006 | *TIP41* | 0.559 |
| 3 | *TIP41* | 0.221 | *UBI3* | 0.007 | *EXP* | 0.644 |
| 4 | *EXP* | 0.253 | *EXP* | 0.008 | *PDS* | 0.683 |
| 5 | *UBI3* | 0.274 | *PDS* | 0.011 | *APT* | 0.690 |
| 6 | *TUB2* | 0.345 | *TUB2* | 0.017 | *ACT* | 0.691 |
| 7 | *EF1α* | 0.399 | *EF1α* | 0.024 | *TUB2* | 0.821 |
| 8 | *APT* | 0.479 | *APT* | 0.026 | *EF1α* | 1.533 |
| **Analysis #05:** Vir1 *x* Avr *x* mock at 24 HPI | | | | | | |
| 1 | *ACT* | 0.106 | *ACT* | 0.002 | *ACT* | 0.890 |
| 2 | *APT* | 0.116 | *TIP41* | 0.003 | *APT* | 0.893 |
| 3 | *EXP* | 0.136 | *APT* | 0.003 | *PDS* | 0.946 |
| 4 | *PDS* | 0.165 | *EXP* | 0.009 | *EXP* | 0.958 |
| 5 | *UBI3* | 0.201 | *TUB2* | 0.011 | *UBI3* | 0.962 |
| 6 | *TIP41* | 0.225 | *UBI3* | 0.011 | *TUB2* | 1.020 |
| 7 | *TUB2* | 0.281 | *PDS* | 0.014 | *TIP41* | 1.097 |
| 8 | *EF1α* | 0.514 | *EF1α* | 0.026 | *EF1α* | 2.430 |
| **Analysis #06:** Vir1 *x* Avr *x* mock at 96 HPI | | | | | | |
| 1 | *TIP41* | 0.108 | *ACT* | 0.003 | *UBI3* | 0.356 |
| 2 | *EXP* | 0.130 | *TIP41* | 0.004 | *TIP41* | 0.509 |
| 3 | *UBI3* | 0.138 | *APT* | 0.004 | *EXP* | 0.521 |
| 4 | *ACT* | 0.259 | *EXP* | 0.009 | *ACT* | 0.609 |
| 5 | *PDS* | 0.289 | *TUB2* | 0.012 | *PDS* | 0.637 |
| 6 | *EF1α* | 0.356 | *UBI3* | 0.012 | *APT* | 0.747 |
| 7 | *TUB2* | 0.426 | *PDS* | 0.015 | *TUB2* | 0.848 |
| 8 | *APT* | 0.531 | *EF1α* | 0.026 | *EF1α* | 1.328 |
| **Analysis #07:** Vir2 *x* Avr *x* mock at 24 HPI | | | | | | |
| 1 | *EXP* | 0.113 | *APT* | 0.002 | *UBI3* | 0.691 |
| 2 | *TIP41* | 0.115 | *ACT* | 0.003 | *APT* | 0.720 |
| 3 | *APT* | 0.127 | *TIP41* | 0.004 | *EXP* | 0.772 |
| 4 | *ACT* | 0.151 | *EXP* | 0.006 | *ACT* | 0.786 |
| 5 | *PDS* | 0.170 | *PDS* | 0.008 | *TIP41* | 0.791 |
| 6 | *UBI3* | 0.194 | *UBI3* | 0.008 | *PDS* | 0.803 |
| 7 | *EF1α* | 0.235 | *TUB2* | 0.011 | *TUB2* | 0.876 |
| 8 | *TUB2* | 0.279 | *EF1α* | 0.018 | *EF1α* | 1.599 |
| **Analysis #08:** Vir2 *x* Avr *x* mock at 96 HPI | | | | | | |
| 1 | *UBI3* | 0.054 | *ACT* | 0.002 | *UBI3* | 0.442 |
| 2 | *PDS* | 0.062 | *TIP41* | 0.004 | *PDS* | 0.533 |
| 3 | *ACT* | 0.068 | *UBI3* | 0.008 | *TIP41* | 0.552 |
| 4 | *TIP41* | 0.116 | *EXP* | 0.009 | *ACT* | 0.569 |
| 5 | *EXP* | 0.187 | *PDS* | 0.009 | *EXP* | 0.661 |
| 6 | *EF1α* | 0.295 | *TUB2* | 0.019 | *APT* | 0.727 |
| 7 | *TUB2* | 0.386 | *EF1α* | 0.024 | *TUB2* | 0.914 |
| 8 | *APT* | 0.499 | *APT* | 0.030 | *EF1α* | 1.459 |
| **Analysis #09:** Vir1 *x* Avr *x* mock at 24 and 96 HPI | | | | | | |
| 1 | *EXP* | 0.193 | *TIP41* | 0.004 | *UBI3* | 0.699 |
| 2 | *UBI3* | 0.200 | *ACT* | 0.004 | *EXP* | 0.736 |
| 3 | *TIP41* | 0.211 | *EXP* | 0.005 | *PDS* | 0.781 |
| 4 | *PDS* | 0.226 | *UBI3* | 0.008 | *ACT* | 0.787 |
| 5 | *ACT* | 0.267 | *PDS* | 0.012 | *TIP41* | 0.827 |
| 6 | *TUB2* | 0.329 | *TUB2* | 0.015 | *TUB2* | 0.911 |
| 7 | *APT* | 0.438 | *APT* | 0.017 | *APT* | 0.924 |
| 8 | *EF1α* | 0.567 | *EF1α* | 0.022 | *EF1α* | 2.009 |
| **Analysis #10:** Vir2 *x* Avr *x* mock at 24 and 96 HPI | | | | | | |
| 1 | *PDS* | 0.132 | *ACT* | 0.002 | *UBI3* | 0.637 |
| 2 | *UBI3* | 0.138 | *TIP41* | 0.004 | *PDS* | 0.668 |
| 3 | *TIP41* | 0.150 | *EXP* | 0.005 | *TIP41* | 0.705 |
| 4 | *ACT* | 0.177 | *UBI3* | 0.009 | *ACT* | 0.721 |
| 5 | *EXP* | 0.210 | *PDS* | 0.009 | *EXP* | 0.773 |
| 6 | *EF1α* | 0.274 | *TUB2* | 0.015 | *APT* | 0.777 |
| 7 | *TUB2* | 0.332 | *APT* | 0.016 | *TUB2* | 0.877 |
| 8 | *APT* | 0.443 | *EF1α* | 0.019 | *EF1α* | 1.617 |
| **Analysis #11:** Vir1 *x* Vir2 *x* mock at 24 HPI | | | | | | |
| 1 | *TUB2* | 0.209 | *TIP41* | 0.004 | *APT* | 1.010 |
| 2 | *TIP41* | 0.236 | *APT* | 0.006 | *UBI3* | 1.049 |
| 3 | *APT* | 0.279 | *TUB2* | 0.008 | *ACT* | 1.100 |
| 4 | *ACT* | 0.320 | *UBI3* | 0.008 | *EXP* | 1.110 |
| 5 | *UBI3* | 0.391 | *ACT* | 0.010 | *PDS* | 1.112 |
| 6 | *EXP* | 0.635 | *EXP* | 0.010 | *TIP41* | 1.214 |
| 7 | *EF1α* | 0.759 | *PDS* | 0.014 | *TUB2* | 1.244 |
| 8 | *PDS* | 0.936 | *EF1α* | 0.029 | *EF1α* | 2.372 |
| **Analysis #12:** Vir1 *x* Vir2 *x* mock at 96 HPI | | | | | | |
| 1 | *EXP* | 0.159 | *TIP41* | 0.005 | *UBI3* | 0.434 |
| 2 | *TUB2* | 0.162 | *EXP* | 0.006 | *TIP41* | 0.635 |
| 3 | *TIP41* | 0.167 | *UBI3* | 0.008 | *EXP* | 0.700 |
| 4 | *UBI3* | 0.226 | *TUB2* | 0.008 | *ACT* | 0.726 |
| 5 | *ACT* | 0.294 | *APT* | 0.009 | *PDS* | 0.754 |
| 6 | *PDS* | 0.323 | *ACT* | 0.010 | *TUB2* | 0.759 |
| 7 | *EF1α* | 0.367 | *PDS* | 0.015 | *APT* | 1.244 |
| 8 | *APT* | 0.426 | *EF1α* | 0.022 | *EF1α* | 1.709 |
| **Analysis #13:** Vir1 *x* Vir2 *x* mock at 24 and 96 HPI | | | | | | |
| 1 | *ACT* | 0.179 | *TIP41* | 0.004 | *UBI3* | 0.844 |
| 2 | *PDS* | 0.183 | *EXP* | 0.004 | *EXP* | 0.967 |
| 3 | *TIP41* | 0.199 | *UBI3* | 0.007 | *PDS* | 0.995 |
| 4 | *UBI3* | 0.219 | *ACT* | 0.007 | *APT* | 1.008 |
| 5 | *TUB2* | 0.236 | *TUB2* | 0.009 | *ACT* | 1.010 |
| 6 | *EXP* | 0.246 | *PDS* | 0.013 | *TUB2* | 1.021 |
| 7 | *APT* | 0.303 | *APT* | 0.013 | *TIP41* | 1.033 |
| 8 | *EF1α* | 0.429 | *EF1α* | 0.026 | *EF1α* | 2.144 |
| **Analysis #14:** Vir1 *x* mock at 24 and 96 HPI | | | | | | |
| 1 | *ACT* | 0.122 | *TIP41* | 0.004 | *UBI3* | 0.863 |
| 2 | *APT* | 0.123 | *EXP* | 0.008 | *EXP* | 0.901 |
| 3 | *PDS* | 0.146 | *UBI3* | 0.008 | *ACT* | 0.999 |
| 4 | *UBI3* | 0.246 | *TUB2* | 0.009 | *TUB2* | 1.006 |
| 5 | *EXP* | 0.266 | *ACT* | 0.009 | *APT* | 1.042 |
| 6 | *TIP41* | 0.280 | *APT* | 0.012 | *PDS* | 1.043 |
| 7 | *TUB2* | 0.298 | *PDS* | 0.015 | *TIP41* | 1.098 |
| 8 | *EF1α* | 0.494 | *EF1α* | 0.032 | *EF1α* | 2.387 |
| **Analysis #15:** Vir2 *x* mock at 24 and 96 HPI | | | | | | |
| 1 | *PDS* | 0.064 | *TIP41* | 0.004 | *UBI3* | 0.708 |
| 2 | *ACT* | 0.068 | *ACT* | 0.005 | *APT* | 0.807 |
| 3 | *UBI3* | 0.078 | *EXP* | 0.006 | *PDS* | 0.851 |
| 4 | *TIP41* | 0.136 | *UBI3* | 0.008 | *TIP41* | 0.874 |
| 5 | *TUB2* | 0.200 | *TUB2* | 0.009 | *ACT* | 0.890 |
| 6 | *EXP* | 0.235 | *PDS* | 0.010 | *EXP* | 0.954 |
| 7 | *EF1α* | 0.291 | *APT* | 0.014 | *TUB2* | 0.995 |
| 8 | *APT* | 0.373 | *EF1α* | 0.021 | *EF1α* | 1.741 |
| **Analysis #16:** Vir1 *x* Vir2 at 24 HPI | | | | | | |
| 1 | *APT* | 0.066 | *TIP41* | 0.004 | *APT* | 0.986 |
| 2 | *TUB2* | 0.073 | *EXP* | 0.008 | *TUB2* | 1.006 |
| 3 | *TIP41* | 0.076 | *APT* | 0.008 | *EXP* | 1.029 |
| 4 | *EXP* | 0.090 | *PDS* | 0.008 | *PDS* | 1.059 |
| 5 | *UBI3* | 0.106 | *UBI3* | 0.012 | *UBI3* | 1.070 |
| 6 | *ACT* | 0.127 | *TUB2* | 0.013 | *ACT* | 1.072 |
| 7 | *PDS* | 0.408 | *ACT* | 0.013 | *TIP41* | 1.095 |
| 8 | *EF1α* | 0.555 | *EF1α* | 0.030 | *EF1α* | 2.455 |
| **Analysis #17:** Vir1 *x* Vir2 at 96HPI | | | | | | |
| 1 | *PDS* | 0.128 | *TIP41* | 0.003 | *UBI3* | 0.388 |
| 2 | *ACT* | 0.138 | *EXP* | 0.005 | *TIP41* | 0.531 |
| 3 | *EXP* | 0.166 | *UBI3* | 0.005 | *EXP* | 0.580 |
| 4 | *TIP41* | 0.191 | *PDS* | 0.008 | *PDS* | 0.664 |
| 5 | *UBI3* | 0.250 | *ACT* | 0.011 | *ACT* | 0.718 |
| 6 | *APT* | 0.328 | *TUB2* | 0.016 | *TUB2* | 0.768 |
| 7 | *EF1α* | 0.364 | *EF1α* | 0.021 | *APT* | 0.822 |
| 8 | *TUB2* | 0.401 | *APT* | 0.029 | *EF1α* | 1.587 |
| **Analysis #18:** Vir1 *x* Vir2 *x* Avr at 24 and 96HPI | | | | | | |
| 1 | *TIP41* | 0.167 | *TIP41* | 0.002 | *UBI3* | 0.757 |
| 2 | *PDS* | 0.174 | *EXP* | 0.005 | *EXP* | 0.808 |
| 3 | *UBI3* | 0.184 | *PDS* | 0.007 | *PDS* | 0.832 |
| 4 | *EXP* | 0.206 | *UBI3* | 0.007 | *TIP41* | 0.859 |
| 5 | *ACT* | 0.241 | *ACT* | 0.010 | *ACT* | 0.879 |
| 6 | *TUB2* | 0.269 | *TUB2* | 0.015 | *TUB2* | 0.889 |
| 7 | *APT* | 0.380 | *APT* | 0.015 | *APT* | 1.020 |
| 8 | *EF1α* | 0.486 | *EF1α* | 0.022 | *EF1α* | 2.020 |
| **Analysis #19:** Vir1 *x* Avr at 24 and 96 HPI | | | | | | |
| 1 | *EXP* | 0.157 | *TIP41* | 0.003 | *UBI3* | 0.732 |
| 2 | *UBI3* | 0.184 | *ACT* | 0.009 | *EXP* | 0.734 |
| 3 | *PDS* | 0.193 | *UBI3* | 0.009 | *PDS* | 0.794 |
| 4 | *TIP41* | 0.211 | *PDS* | 0.009 | *ACT* | 0.816 |
| 5 | *ACT* | 0.271 | *EXP* | 0.012 | *TUB2* | 0.822 |
| 6 | *TUB2* | 0.300 | *TUB2* | 0.017 | *TIP41* | 0.836 |
| 7 | *APT* | 0.429 | *EF1α* | 0.026 | *APT* | 1.047 |
| 8 | *EF1α* | 0.576 | *APT* | 0.035 | *EF1α* | 2.137 |
| **Analysis #20:** Vir2 *x* Avr at 24 and 96 HPI | | | | | | |
| 1 | *PDS* | 0.097 | *TIP41* | 0.002 | *PDS* | 0.645 |
| 2 | *TIP41* | 0.104 | *ACT* | 0.006 | *UBI3* | 0.657 |
| 3 | *UBI3* | 0.115 | *PDS* | 0.006 | *TIP41* | 0.681 |
| 4 | *ACT* | 0.206 | *EXP* | 0.009 | *EXP* | 0.705 |
| 5 | *EXP* | 0.206 | *UBI3* | 0.010 | *ACT* | 0.723 |
| 6 | *TUB2* | 0.255 | *TUB2* | 0.016 | *TUB2* | 0.778 |
| 7 | *EF1α* | 0.311 | *EF1α* | 0.020 | *APT* | 0.854 |
| 8 | *APT* | 0.437 | *APT* | 0.035 | *EF1α* | 1.683 |

^1^M = average expression stability, calculated by geNorm algorithm in the qBase software.

^2^Stability value = stability of gene expression calculated by intra and intergroup variation by NormFinder algorithm.

^3^Std dev [±CP] = standard deviation in BestKeeper algorithm, significant values < 1.0. All genes showed significant p-value (= 0.001) by Pearson's correlation test.
