# Supplementary material for: Stability analysis of reference genes for RT-qPCR assays involving compatible and incompatible Ralstonia solanacearum-tomato ‘Hawaii 7996’ interactions
Source: Sci Rep. 2021 Sep 21;11:18719. doi: 10.1038/s41598-021-97854-8 (PMC8455670; doi:10.1038/s41598-021-97854-8)
Supplement: Supplementary file 3 — Supplementary Figure S1. [file 41598_2021_97854_MOESM3_ESM.docx]

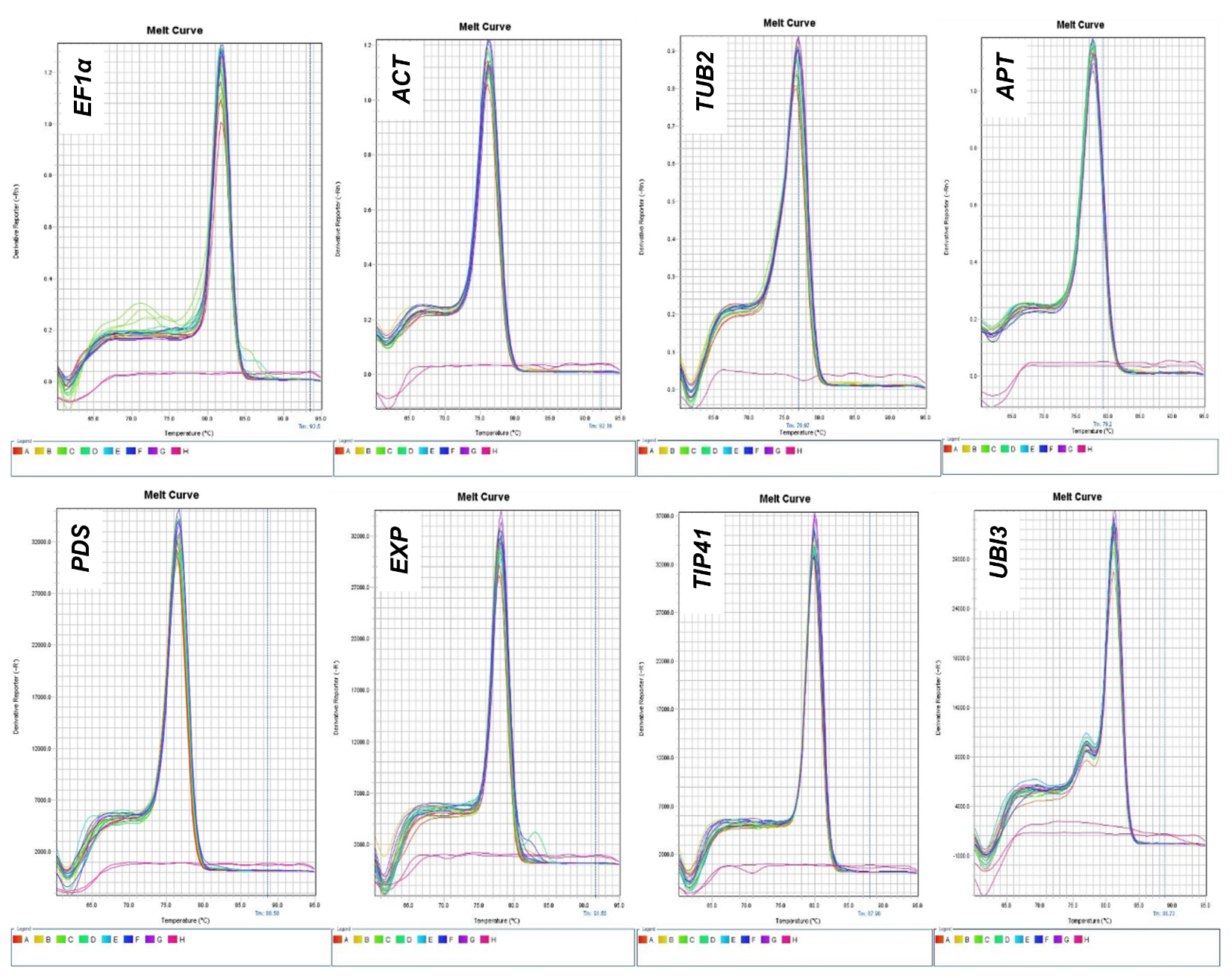


**Supplementary Figure S1.** **Melting curves of the eight tomato genes/alleles [*viz*. primers for the genes actin (*ACT*), adenine–phosphoribosyl–transferase 1 (*APT*), β–2–tubulin (*TUB2*), elongation factor 1–alpha (*EF1α*), the *Arabidopsis thaliana* expressed protein (*EXP*), TIP41–interacting protein (*TIP41*), phytoene desaturase (*PDS*), and ubiquitin (*UBI3* = *UBQ*)] employed in the assays involving the pathosystem tomato ‘Hawaii 7996’ and compatible/virulent and incompatible/avirulent *Ralstonia solanacearum* isolates at different sampling collection times.** PCR assay specificity was measured by the presence of unique amplicons using melting curve analysis, with data obtained from three technical replicates of different cDNA samples. No amplification was observed in negative controls lacking template cDNA.
