# Supplementary material for: Stability analysis of reference genes for RT-qPCR assays involving compatible and incompatible Ralstonia solanacearum-tomato ‘Hawaii 7996’ interactions
Source: Sci Rep. 2021 Sep 21;11:18719. doi: 10.1038/s41598-021-97854-8 (PMC8455670; doi:10.1038/s41598-021-97854-8)
Supplement: Supplementary file 4 — Supplementary Figure S2. [file 41598_2021_97854_MOESM4_ESM.docx]

**
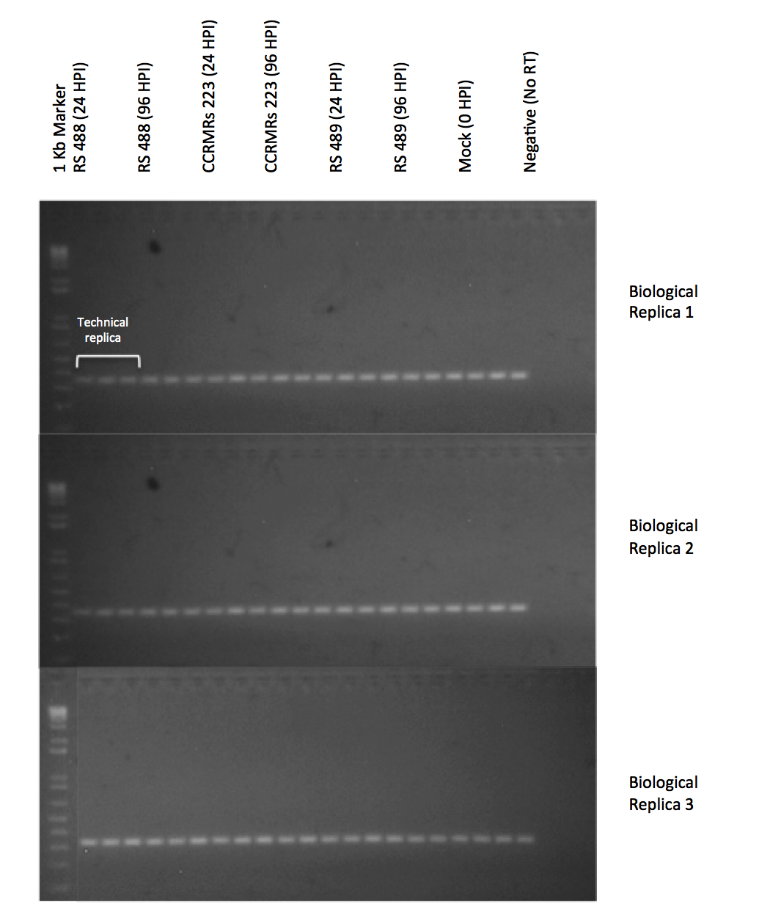
**

**Supplementary Figure S2.** Full length agarose gel displaying single amplicons obtained for the *UBI3* gene following RT-qPCR with the primers described in Table 2 and staining with UniSafe Dye^®^. Each sample comprised three technical replicates and each set of samples comprised three biological replicates
